# Convalescent Plasma: A Potential Life-Saving Therapy for Coronavirus Disease 2019 (COVID-19)

**DOI:** 10.3389/fpubh.2020.00437

**Published:** 2020-08-06

**Authors:** Ahmed N. Alghamdi, Ahmed S. Abdel-Moneim

**Affiliations:** ^1^Microbiology Department, College of Medicine, Taif University, Al-Taif, Saudi Arabia; ^2^Virology Department, Faculty of Veterinary Medicine, Beni-Suef University, Beni Suef, Egypt

**Keywords:** COVID-19, SARS-CoV-2, coronavirus, plasma therapy, immunetherapy

## Introduction

The last two decades witnessed the emergence of three zoonotic coronaviruses that crossed the species barrier and caused outbreaks in humans: severe acute respiratory syndrome coronavirus (SARS-CoV) in 2002, Middle East respiratory syndrome coronavirus (MERS-CoV) in 2012, and most recently, severe acute respiratory syndrome coronavirus-2 (SARS-CoV-2) in 2019 (1). All three viruses are β-coronaviruses belonging to the *Orthocoronavirinae* subfamily in the *Coronaviriade* viral family (1). Similar to SARS-CoV, the newly emerged SARS-CoV-2 utilizes the angiotensin-converting enzyme 2 (ACE2) cellular receptor for cell entry (2). Although many drugs have been proposed as potential therapeutic agents for coronavirus disease 2019 (COVID-19), no specific antiviral agent has been proven effective to date (3). Convalescent plasma is effective in treating many viral conditions, including respiratory infections (4). Hence, the therapeutic potential of convalescent plasma for COVID-19 is a noteworthy topic. This viewpoint discusses the plausibility of using convalescent plasma from COVID-19 recovered patients as an effective, feasible therapeutic intervention for COVID-19.

## History

Convalescent plasma has been used since the 1890s to treat several infectious diseases (5). In the early 20th century, convalescent sera obtained from recovered individuals during outbreaks of viral etiology, such as influenza and measles, were used for therapeutic purposes (5). In the early 21st century, convalescent plasma was utilized to increase the survival rate among critically ill patients during the H1N1 pandemic in 2009, as well as during the SARS-CoV and MERS-CoV outbreaks in 2002 and 2012, respectively (5). Thirty-two studies demonstrated a consistent reduction in mortality among convalescent plasma-treated patients with severe SARS and influenza infections without convalescent plasma-related adverse effects (6). The pooled data from 27 out of these 32 studies revealed a statistically significant reduction in the pooled odds of death among the convalescent plasma-treated group compared with the control group (6). Further, during the Ebola virus epidemic in West Africa in 2014, convalescent plasma therapy (CPT) was used empirically, as recommended by the World Health Organization (7).

## Mode of Action

Convalescent plasma exemplifies passive immune therapy, which combats invading pathogens by administering antibodies (2). It is hypothesized that polyclonal antibodies in convalescent plasma neutralize the circulating initial inoculum of microbes and facilitate antibody-dependent cellular cytotoxicity and phagocytosis (5). In the context of CPT for COVID-19, the antibodies are anticipated to employ both immune mechanisms, but the main one would be neutralization, which occurs when neutralizing antibodies block SARS-CoV-2 spike proteins, thereby aborting viral entry (2, 4, 5). Hosts naturally develop antibodies 10–14 days post-infection; therefore, convalescent plasma administration before seroconversion is believed to be more therapeutically effective (4, 5).

## CPT in Treating Emerging Coronaviruses

### CPT for SARS and MERS

Several studies have shown a favorable outcome of CPT in treating infections caused by emerging coronaviruses (3, 5, 7–10). A retrospective non-randomized comparison study addressed the outcome of 40 severely affected SARS-CoV patients, where the treatment group (*n* = 19) received SARS convalescent plasma while the control group was kept only on methylprednisolone after both groups had finished an empirical combination of ribavirin and methylprednisolone (8). Patients in the treatment group were given 200–400 ml of convalescent plasma obtained using an apheresis device from SARS-recovered individuals with a SARS-IgG titer ranging from 160 to 2,560 (8). In 2003, researchers addressed the outcome in 80 SARS-CoV-infected patients on convalescent plasma (7). The outcome was deemed to be good if the hospital admission lasted <23 days post-onset. The SARS-CoV convalescent plasma volume used in this study ranged from 160 to 640 ml with a SARS-IgG titer ranging from 160 to 2,560 (7). The mortality rate among those 80 critical SARS patients was 12.5%, while the overall mortality rate when the SARS epidemic struck Hong Kong in 2003 was 17% (8). Moreover, this study found that convalescent plasma administration before the 14th day post-onset is associated with a better outcome than its administration after this point (58.3 vs. 15.6%). It was also evident from this study that convalescent plasma administration in SARS-CoV PCR-positive but SARS-seronegative patients was more therapeutically effective than in SARS-CoV PCR-positive and SARS-seropositive patients (66.7 vs. 20%) (7). These findings are in accordance with the notion that the effectiveness of CPT is directly related to its early administration prior to seroconversion (3, 5, 8). A study involving three critical SARS-CoV-infected patients who were treated with 500 ml SARS-CoV convalescent plasma of SARS-CoV IgG titer >640 infusion each showed clinical improvement followed by viral burden decline (9). CPT was used to treat three critically ill MERS cases in South Korea (10). The study concluded that effective convalescent plasma treatment was associated with a MERS-IgG titer ≥80, while an IgG titer of 40 was ineffective (10). Further, the neutralization activity could be predicted by ELISA-IgG without conducting sophisticated BSL-3 laboratory-dependent procedures, such as the plaque reduction neutralization test. At a cutoff optical density of 1.6 and 1.9, the specificity of ELISA-IgG in predicting the neutralization activity was ≥95 and 100%, respectively (10).

### CPT for COVID-19

In an uncontrolled case series, five ARDS-complicated COVID-19 patients were on mechanical ventilation, four of whom were ≥50 years of age. They received 400 ml of convalescent plasma infusion each immediately after being obtained by apheresis from ABO-compatible donors (3). The convalescent plasma had an IgG titer >1000 and a neutralization titer >40. Following plasma transfusion, all patients manifested a restored normal body temperature within 3 days and the range of their PaO_2_/FiO_2_ improved from 172–276 to 284–366 within 12 days (3) (Table 1). Further, viral load and inflammatory cytokines started to decline while serological responses began to mount after the CPT. Moreover, three out of the five patients were extubated and discharged (3). Anecdotal pieces of evidence on the safety and efficacy of convalescent plasma in treating COVID-19 have been reported (11). After infusion with 200 ml volume and ≥640 neutralization titer convalescent plasma, 10 critical COVID-19 patients on supportive and antiviral treatments improved in terms of clinical and laboratory parameters (11). Post-plasma infusion, fever, and respiratory symptoms subsided within 3 days while RNAemia took 6 days to become undetected. No serious adverse reactions were reported (11) (Table 1). Another four severe SARS-CoV-2-infected cases on supportive and antiviral therapy improved following convalescent plasma administration with no adverse effects. The volume of CPT ranged from 200 to 2,400 ml (12) (Table 1). The first reported use of CPT for COVID-19 in South Korea was on two ARDS-complicated SARS-CoV-2-infected patients (13). Convalescent plasma was obtained from two fully recovered SARS-CoV-2-causing pneumonia cases with anti-SARS-CoV-2 ELISA optical density of 0.586 and 0.532 (cutoff: 0.22). Despite being on lopinavir/ritonavir and hydroxychloroquine, both CPT recipients had been suffering from a worsening course of ARDS-complicated SARS-CoV-2 infection (PaO_2_/FiO_2_: < 100) prior to the convalescent plasma infusion. After 500 ml of convalescent plasma infusion each, both critically ill COVID-19 patients improved in terms of symptoms and infection-related markers, with no adverse effects reported. Their escalating viral loads prior to the plasma administration started to dramatically fall the next day after the convalescent plasma infusion, while their oxygen demand gradually decreased until they were successfully extubated. Although the subjects in this study received methylprednisolone within 2 days prior to convalescent plasma infusion, their viral burden decreased afterwards, suggesting the successful neutralization effect of the administered plasma (13) (Table 1). A recent study in Wuhan, China, described the efficacy and safety of CPT on six COVID-19 cases (14). At least 200 ml of convalescent plasma infusion was administered to six laboratory-confirmed COVID-19 cases, five of whom had lower respiratory tract involvement. All convalescent plasma recipients showed clinical improvement without any adverse effects. Yeh et al. linked CPT in COVID-19 to radiological and serological improvements in terms of resolution of COVID-19-related abnormal radiological findings and mounting numbers of anti-SARS-CoV-2 antibodies, respectively (Table 1). There was evidence of the clinical benefits of convalescent plasma in those running a late course of COVID-19 even after seroconversion exists (14). Hence, the efficacy of convalescent plasma in relation to seroconversion should be rigorously evaluated. CPT succeeded in lowering the SARS-CoV-2 viral burden, although it was administered after steroids (3, 13), contradicting the general notion that steroids have a counteractive effect on CPT (11). A thorough assessment of the relationship between steroids and CPT may lead to a more effective therapeutic combination. As of this writing, more than 80 clinical trials are aiming to investigate the safety and efficacy of CPT in COVID-19 subjects; however, results have yet to be posted (15). The scarcity of convalescent plasma donors should not be a problem, with millions of fully recovered COVID-19 cases all over the globe.

**Table 1:** Safety and efficacy of convalescent plasma therapy for COVID-19 infected patients.

**Study**	**Participants**	**Pre-CPT status**	**CPT**	**Post-CPT outcome**	**Adverse Effects**	**References**
1	n:5, 3♂:2♀, age (36–65 y.), HTN & MR (n:1)	ARDS-complicated COVID-19 (n:5), MV (n:5), ECMO (n:1), PaO_2_/FiO_2_ (range, 172–276), neutralizing Ab (range, 40–160)	Volume (400 ml), neutralizing Ab titer (range, 80–480), administration day (range, 10–22 d. post-admission)	Fever subsided within 3 d. post-CPT (n:5), viral load decline (undetectable within 12 d. post-CPT) (n:5), PaO_2_/FiO_2_ improved within 12 d. post-CPT (range, 284–366) (n:5), neutralizing Ab titer increased (range, 80–320 and 160–480 at 1st and 3rd d post-CPT, respectively), radiological improvements noticed from the 3rd d post-CPT (n:5), MV removal within 12 d. post-CPT (n:3), ECMO removal within 5 d. post-CPT (n:1), patient discharge (n:3)	None (n:5)	(3)
2	n:10, 6♂:4♀, age (34–78 y.), HTN (n:3), cardiac and cerebrovascular diseases (n:1)	Severe COVID-19 (n:10), MV (n:3), high-flow O_2_ (n:3), low-flow O_2_ (n:2), SaO_2_% (median,93; range, 89–96.5), neutralizing Ab titer (range, 160–640)*	Volume (200 ml), neutralizing Ab titer (≥640), administration day (range, 10–20 d. post-onset)	Symptoms and SaO_2_% (median,96; range, 95–96.5) both improved within 3 d post-CPT (n:10), viral load decline (undetectable within 6 d. post-CPT)‡ (n:7), neutralizing Ab titer increased to 640*† (n:5), radiological improvements noticed within 3 d post-CPT (n:10), MV removal (n:2), high-flow O_2_ not needed anymore (n:2), patient discharge (n:3), patient improved and ready for discharge (n:7)	Evanescent facial red spots (n:1), none (n:9)	(11)
3	n:4, 2♂:2♀, Age (31–73 y.), COPD (n:1), HTN (n:1), HTN and CKD (n:1), pregnant (n:1)	ARDS-complicated COVID-19 (n:4), MV (n:3), non-invasive ventilation and high-flow O_2_ (n:1), ECMO (n:1)	Volume (range, 200–2,400 ml), administration day (range, 12–19 d post-admission)	Clinical and radiological improvement (n:4), viral load decline within 30 d. post-CPT^§^ (qRT-PCR undetectability range, 6–30 d. post-CPT) (n:4), MV removal within 20 d. post-CPT^§^ (n:2), ECMO removal within 7 days post-CPT^§^ (n:1), Patient discharge (n:3)	None (n:4)	(12)
4	n:2, 1♂:1♀, Age (67 and 71 y.), HTN (n:1)	ARDS-complicated COVID-19, MV (n:2), PaO_2_/FiO_2_: 86 and 76	Volume: 500 ml, Anti-SARS-2 IgG ELISA: 0.586 and 0.532 (Cut-off: 0.22), administration day: 6th and 10th d post-admission	Clinical and radiological improvement (n:2), viral load decline (undetectable in both patients after 16 and 14 d post-CPT, respectively), PaO_2_/FiO_2_ in both patients increased to 300 and 230 within 8 and 6 d post-CPT, respectively, MV removal (n:2), patient discharge (n:1)	None (n:2)	(13)
5	n:6, 3♂:3♀, Age (range, 28–75 y.), Sjören syndrome (n:1)	COVID-19 (n:6), clinical and radiological picture of SARS-CoV-2 causing LRTI^Ω^ (n:5)	Volume (range, 200–600 ml), administration day (33–50 d. post-onset)	Clinical and radiological improvement^Ω^ (n:5), viral load decline and eventually undetectable* (n:5), patient discharge* (n:5)	None (n:6)	(14)

*HTN, Hypertension; MR, Mitral regurgitation; ARDS, Acute respiratory distress syndrome; MV, Mechanical ventilation; ECMO, Extracorporeal membrane oxygenation; IFN-α, Interferon-alpha; COPD, Chronic obstructive pulmonary disease; CKD, Chronic kidney disease; LRTI, Lower respiratory tract infection*.

* *One patient's data was unavailable*.

† *Four patients' neutralizing Ab titer remained the same as before the CPT at 640*.

‡ *Three patients had already had undetectable SARS-CoV-2 prior to the CPT*.

§ *From the first or the only CPT dose received*.

Ω *One patient was SARS-CoV-2 positive and asymptomatic during the CPT after being previously symptomatic with no LRTI involvement*.

## Drawbacks of Using CPT

The drawbacks associated with CPT include adverse effects, such as transfusion transmissible infections (TTIs) and transfusion-related acute lung injury (TRALI) (2, 5). In addition, its effectivity depends on the neutralization titer (2, 10). However, based on available studies, plasma infusion is a safe medical practice, mainly due to advances in blood banking and transfusion, including ABO compatibility checking and TTI screening and monitoring during and after transfusion (2, 5). Further, in a reported case of TRALI following MERS convalescent plasma infusion, neither anti-human leukocyte antigen nor anti-human neutrophil antigen, both of which are TRALI pathophysiology key players, were detected in the donated plasma (2, 16). Additionally, neutralization activity could be predicted by ELISA-IgG (10).

## Conclusion

Currently, no specific antiviral agent has been proven for SARS-CoV-2 infection. However, based on available data, it is plausible to consider CPT as an effective, safe, and feasible therapeutic option for COVID-19. Determining the effective dose of convalescent plasma infusion is essential, along with other variables such as the neutralization titer.

## Author Contributions

ASA contributed conception of the study and critically revised the manuscript. ANA wrote the first draft of the manuscript. All authors contributed to manuscript revision, read, and approved the submitted version.

## Conflict of Interest

The authors declare that the research was conducted in the absence of any commercial or financial relationships that could be construed as a potential conflict of interest.
